# *DREB1* and *DREB2* Genes in Garlic (*Allium sativum* L.): Genome-Wide Identification, Characterization, and Stress Response

**DOI:** 10.3390/plants12132538

**Published:** 2023-07-03

**Authors:** Mikhail A. Filyushin, Olga K. Anisimova, Anna V. Shchennikova, Elena Z. Kochieva

**Affiliations:** Research Center of Biotechnology, Institute of Bioengineering, Russian Academy of Sciences, Leninsky Ave. 33, Bld. 2, Moscow 119071, Russia; lelikanis@yandex.ru (O.K.A.); shchennikova@yandex.ru (A.V.S.); ekochieva@yandex.ru (E.Z.K.)

**Keywords:** *Allium sativum* L., DREB proteins, gene structure, gene expression, cold stress, *Fusarium*

## Abstract

Dehydration-responsive element-binding (DREB) transcription factors (TFs) of the A1 and A2 subfamilies involved in plant stress responses have not yet been reported in *Allium* species. In this study, we used bioinformatics and comparative transcriptomics to identify and characterize *DREB* A1 and A2 genes redundant in garlic (*Allium sativum* L.) and analyze their expression in *A. sativum* cultivars differing in the sensitivity to cold and *Fusarium* infection. Eight A1 (*AsaDREB1.1–1.8*) and eight A2 (*AsaDREB2.1–2.8*) genes were identified. *AsaDREB1.1–1.8* genes located in tandem on chromosome 1 had similar expression patterns, suggesting functional redundancy. *AsaDREB2.1–2.8* were scattered on different chromosomes and had organ- and genotype-specific expressions. *AsaDREB1* and *AsaDREB2* promoters contained 7 and 9 hormone- and stress-responsive *cis*-regulatory elements, respectively, and 13 sites associated with TF binding and plant development. In both *Fusarium*-resistant and -sensitive cultivars, fungal infection upregulated the *AsaDREB1.1–1.5*, *1.8*, *2.2*, *2.6*, and *2.8* genes and downregulated *AsaDREB2.5*, but the magnitude of response depended on the infection susceptibility of the cultivar. Cold exposure strongly upregulated the *AsaDREB1* genes, but downregulated most *AsaDREB2* genes. Our results provide the foundation for further functional analysis of the DREB TFs in *Allium* crops and could contribute to the breeding of stress-tolerant varieties.

## 1. Introduction

Garlic (*Allium sativum* L.) is an economically important vegetable crop belonging to the most numerous (over 920 species) *Allium* genus of monocotyledons [1,2,3]. The *A. sativum* bulbs and green parts are used as spices and as traditional remedies for various diseases because of their anticarcinogenic, antioxidant, antidiabetic, renoprotective, antiatherosclerotic, antibacterial, antifungal, and antihypertensive properties [4]. In addition, garlic and other *Allium* plants are in demand for intercropping and crop rotation as they improve soil quality and reduce soil fungal communities, including *Fusarium* [5,6,7,8,9]. The versatile use of *Allium* spp. suggests their high adaptability to various environmental conditions.

*A. sativum* originated about 10,000–5000 years ago in the Mediterranean region and Western and Central Asia; since then, this species has undergone long-time artificial selection and is currently cultivated all over the world [1,3], and some researchers suggest that most of the available *A. sativum* genotypes have emerged as a result of mutations in single clones [2]. However, despite the lack of sexual reproduction, garlic is characterized by a high phenotypic diversity [3,10,11]. As a result, *A. sativum* demonstrates good adaptability to various stresses, among which ambient temperature changes and fungal infections are considered to be important factors affecting garlic development and yield [3,12,13,14,15].

Garlic, with its shallow root system [12], is sensitive to low temperatures, which, on the one hand, stimulate sprouting, flowering, and bulb initiation, and on the other, have negative effects on plant development [13,14]. However, the data on the genetic mechanisms regulating the response of garlic (as well as other *Allium* species) to low temperatures are limited. A previous study on the Korean garlic transcriptome identified 14 genes differentially expressed in response to cold, including those encoding pathogenesis-related (PR) protein 1, alliinase, the oxidoreductase family, cell-wall associated enzymes, a transcription factor (TF) of the HMG family, and proteins involved in gibberellin regulation and chlorophyll a/b binding [13]. Comparative transcriptomics of onion *(Allium cepa*) exposed to cold and freezing has revealed the induction of genes encoding a CBL-interacting protein kinase, heat shock proteins, and chitinases, as well as TFs of the DREB, MYB, CBL, bZIP, ZAT, HSPs, and bHLH families [16].

Garlic is susceptible to infections caused by *Fusarium* spp. and are considered its most harmful fungal pathogens; among them, *F. proliferatum* is responsible for *Fusarium* basal rot (FBR) of the bulbs and wilt of the leaves [15,17]. A comparison of the recently assembled *A. sativum* (cv. Ershuizao) reference genome and transcriptome [1] with those of other garlic varieties indicates that *Fusarium* infection affects the expression of SWEET sugar uniporters [18] and PR factors such as β-1,3-glucanases and Barwin-domain, CAP-domain, and thaumatin-like proteins [17,19,20].

In plants, stress response is controlled by phytohormones. Among them, salicylic acid (SA), jasmonic acid (JA), and ethylene (ET) are mostly involved in reactions to biotic stresses, including various infections [21], whereas abscisic acid (ABA) along with auxins, brassinosteroids, cytokinins, ET, gibberellic acid (GA), JA, and strigolactones regulate the response to abiotic stresses such as drought, salt, cold, and heat [22]; however, it is also involved in the plant response to pathogens [21]. Stress-responsive hormone-mediated signaling is under the control of TFs, particularly the NAC, MYB, WRKY, bZIP, and AP2/ERF families [23]. The latter includes dehydration-responsive element-binding (*DREB*) proteins A1–A6, among which A1 (*DREB1*/C-repeat factor (CBF)) and A2 (*DREB2*) are the largest subfamilies known to be closely involved in the response to abiotic stresses, as evidenced by their differential expression after treatment with ABA [23,24,25,26,27,28,29]. Thus, *DREB1*/CBF proteins play an important role in the regulation of cold and frost tolerance [23,24,25,26,27], whereas *DREB2* TFs are involved in the response to a wider range of environmental factors, including drought, salt, osmotic stress, heavy metals, and extreme temperatures [23,28,30,31]. Furthermore, it is possible that DREB TFs could regulate the response to pathogens. There is evidence that the *Arabidopsis DEAR1* (*DREB and ear motif protein 1*) gene, highly homologous to *DREB1*/*CBF,* is activated by pathogen infection because its overexpression leads to the upregulation of PR genes, repression of defense negative regulators, suppression of *Pseudomonas syringae* growth, and increased resistance to *Botrytis cinerea* [32,33]. In sugarcane (*Saccharum officinarum* L.), the overexpression of *Ea-DREB2B* (A2 type) positively affects the rhizosphere microbiome [34], whereas in coriander (*Coriandrum sativum* L.), *DREB* genes are differentially expressed in response to *Protomyces macrosporus* infection [35]. However, in the order Asparagales, which includes the *Allium* genus (Amaryllidaceae), *DREB* TFs have been reported only in *Dendrobium catenatum* Lindl. (Orchidaceae) [36], and there are no reports on the *DREB* genes in garlic or other *Allium* spp.

In this study, we identified and characterized the *A. sativum* genes belonging to the *DREB1* and *DREB2* subfamilies and analyzed their expression in different garlic tissues. To determine the role of *DREB1* and *DREB2* genes in garlic anti-stress defense, we investigated their transcriptional response to cold and *F. proliferatum* infection in *A. sativum* cultivars differing in sensitivity to FBR and winter hardiness. Our results provide the information on the *DREB* genes in *A. sativum*, which can be used in the breeding of stress-tolerant garlic varieties.

## 2. Results

### 2.1. Identification and Characterization of DREB1 and DREB2 Genes in the A. sativum cv. Ershuizao Genome

Sixteen sequences of full-length *DREB* genes were identified by in silico analysis of the *A. sativum* cv. Ershuizao genome (PRJNA606385) and transcriptome (PRJNA607255), and were annotated as *A. sativum* (*Asa*) *DREB1.1–1.8* and *DREB2.1–2.8* (Table 1). Given the lack of data on *DREB* genes in *Allium* spp., the gene numbering was based on the order of their chromosomal location, according to previous studies [17,19,20].

**Figure 1:**
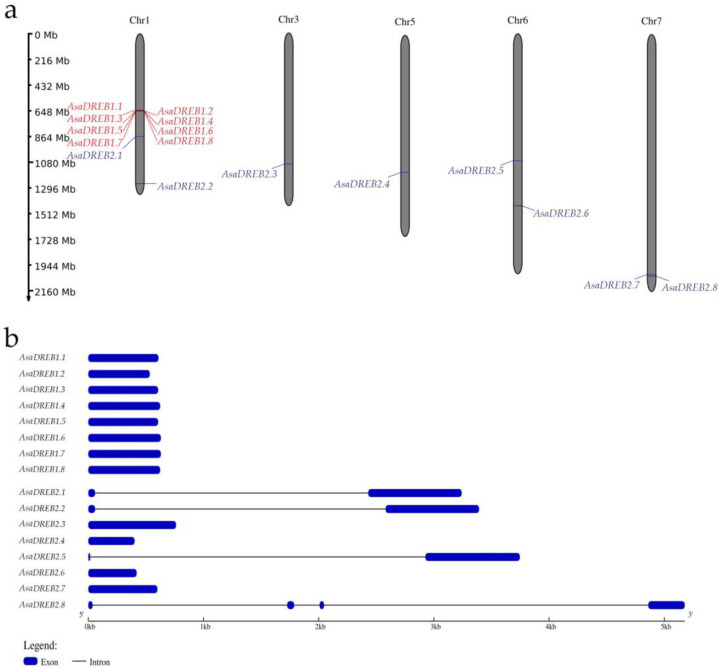
Distribution and structure of the *AsaDREB* genes identified in the *A. sativum* genome. (**a**) Chromosomal localization of *AsaDREB1.1–1.8* (red) and *AsaDREB2.1–2.8* (blue) genes. (**b**) Exon-intron composition. Chromosome lengths are based on the *A. sativum* cv. Ershuizao genome (PRJNA606385); chr, chromosome.

All *AsaDREB1* genes were tandemly located on chromosome 1, whereas *AsaDREB2* genes were identified on several chromosomes: *AsaDREB2.1–2.6* genes were evenly distributed over chromosomes 1, 3, 5, and 6, and *AsaDREB2.7* and *2.8* formed a tandem cluster on chromosome 7 (Figure 1a).

All *AsaDREB1* and half of *AsaDREB2* genes (*AsaDREB2.3*, *2.4*, *2.6*, and *2.7*) were intronless, whereas *AsaDREB2.1*, *2.2*, *2.5*, and *2.8* contained 1–3 introns (Table 1, Figure 1b). No splice variants were found.

### 2.2. Characterization of Putative AsaDREB1 and AsaDREB2 Proteins

Putative *AsaDREB1.1–1.8* and *AsaDREB2.1–2.8* proteins contained 177–209 and 133–289 amino acids (aa), respectively; their molecular weight (MW) and isoelectric point (pI) values are shown in Table 1. The secondary structure of all *AsaDREB* proteins contained a functional DNA-binding APETALA2 (AP2) domain of 60 aa (NCBI accession: cl00033; pfam00847) (Supplementary Figure S1a); InterPro domains: DNA-binding, integrase-type (IPR016177) and pathogenesis-related transcriptional factor/ERF, DNA-binding (IPR001471).

In the *AsaDREB1* group, all proteins (except for *AsaDREB1.2*) had the AP2 domain framed with the PKRR/SAGRTKFRETRHF and DSPR/H consensuses (Supplementary Figure S1a), corresponding to the nuclear localization signal (NLS) P/KKR/KP/RA/TGRT/KKFRETRHP and the DSAW motif, respectively, previously found in *DREB1* TFs [37]. The *AsaDREB1* C-terminus contained the LWS/TY/F motif previously reported in the C-terminal 98-aa transactivation domain [38].

*AsaDREB2* proteins contained a highly conserved GKGGPxN motif, which was a part of the NLS sequence upstream of the AP2 domain (Supplementary Figure S1b) and which is common for *DREB2* TFs of other plant species [38].

Gene Ontology (GO) analysis indicated that all identified *AsaDREB* proteins had nuclear localization and could regulate gene transcription (GO:0006355). In addition, some *AsaDREB2* factors were predicted to be involved in defense response and ET-activated signaling (*AsaDREB2.1*, GO:0006952 and GO:0009873), heat acclimation (*AsaDREB2.4* and *AsaDREB2.8*, GO:0010286), and positive regulation of nucleic acid-templated transcription (*AsaDREB2.5* and *AsaDREB2.8*, GO:1903508).

**Figure 2:**
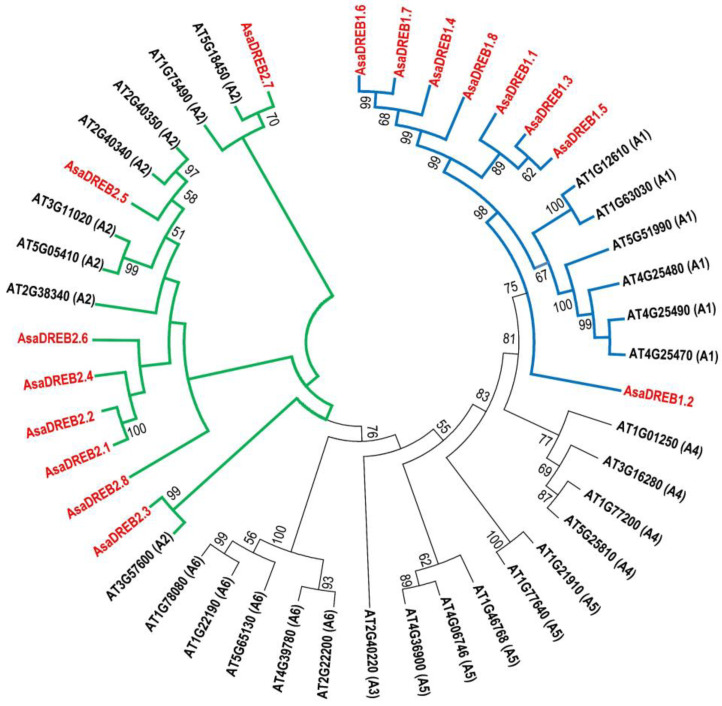
Evolutionary relationships between *AsaDREB1* and *AsaDREB2* proteins (red) and *A. thaliana* DREB TFs (black; NCBI ID and A-type are indicated). The unrooted dendrogram was constructed using the Neighbor-Joining method (bootstrap test: 1000 replicates) in MEGA 7.0.26; the evolutionary distances were computed using the JTT matrix-based method and are in the units of the number of amino acid substitutions per site.

Phylogenetic analysis confirmed the distribution of the *AsaDREB* factors into A1 and A2 groups distinct from the clusters formed by *Arabidopsis thaliana* (AT) DREB A3, A4, A5, and A6 (Figure 2). *AsaDREB1.2* made the basis of the A1 clade, whereas the remaining seven *AsaDREB1* and *Arabidopsis DREB1* proteins showed species-specific clustering. In contrast, clade A2 proteins formed three subgroups: (1) *AsaDREB2.7* clustered with AT5G18450 and AT1G75490; (2) *AsaDREB2.8* was at the basis of a subgroup including *AsaDREB2.5*, AT2G40340, and AT2G40350 and *AsaDREB2.1*/*2.2*/*2.4*/*2.6*; and (3) *AsaDREB2.3* clustered with AT3G57600 (Figure 2).

As *A. thaliana* is a dicot plant, we included *DREB1* and *DREB2* proteins of a monocot *Zea mays* L. (Zm) in the phylogenetic analysis. This addition did not affect the separate clustering of *AsaDREB1* proteins, but allowed for the determination of putative maize orthologs (ZmDREB2.4–2.6) for *AsaDREB2.3* (high bootstrap values), whereas *AsaDREB2.5*, *2.7*, and the *AsaDREB2.1*/*2.2*/*2.4*/*2.6*/*2.8* group formed distinct branches in which any homology was supported by only low bootstrap values (Supplementary Figure S2).

**Figure 3:**
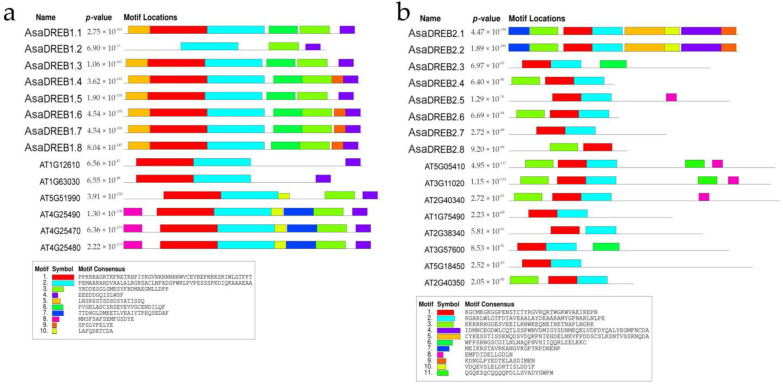
Distribution of conserved motifs in *A. sativum* and *A. thaliana DREB1* (**a**) and *DREB2* (**b**) proteins. Analysis was performed using MEME 5.4.1; the length of each box corresponds to that of the motif.

The phylogenetic division was supported by MEME-based analysis, which revealed 10 and 11 conserved motifs in the *AsaDREB1*/*ATDREB1* and *AsaDREB2*/*ATDREB2* sets, respectively (Figure 3). *AsaDREB1* proteins had a highly conserved motif arrangement, whereas *AsaDREB2* proteins showed at least two distinct motif patterns; in both groups, motifs 1 and 2 constituted NLS together with the AP2 domain.

All *AsaDREB1* factors contained motifs 2–4, of which most (except for *AsaDREB1.2*) had motifs 1, 5, and 6 and half (*AsaDREB1.4* and *1.6–1.8*) had motif 9. *AsaDREB1* differed from ATDREB1 in the absence of motifs 7, 8, and 10 and the presence of motifs 5, 6, and 9 (Figure 3a). Thus, motifs 1–4 could be considered common for *DREB1* proteins of *A. sativum* and *A. thaliana*.

All *AsaDREB2* contained motif 1, of which most of them (except *AsaDREB2.8*) had motif 2 and many (*AsaDREB2.1*, *2.2*, *2.4*, *2.6*, and *2.8*) had motif 3. *AsaDREB2.1* and *2.2* significantly differed from the other proteins of the *DREB2* group by the presence of motifs 4, 5, 7, 9, and 10. Motif 8 was unique for *AsaDREB2.5* and three *ATDREB2* proteins, and motif 11 was found only in two *ATDREB2* proteins and was absent in all *AsaDREB2* proteins (Figure 3b). Thus, motifs 1–3 and 8 could be considered common for *DREB2* factors of *A. sativum* and *A. thaliana*.

### 2.3. Analysis of AsaDREB1 and AsaDREB2 Promoter Regions

Considering the role of *DREB* genes in plant development and stress response, we searched for *cis*-acting elements in the 5′ untranslated regions (UTRs) and promoters (~1 kb upstream of the start codon). As a result, 7 hormone- and 9 stress-responsive elements and 13 sites associated with TF binding, meristem development, cell cycle regulation, and plant reproduction were identified (Table 2).

The most common elements were ABA-responsive ABRE motifs (lacking only in *AsaDREB1.7* and *AsaDREB1.8*) and MYB TF binding sites (present in all of the identified genes), which were closely related to the ABA-mediated plant stress response [39]. Promoters of the *AsaDREB1.1*, *1.3*, *1.5*, *2.5*, and *2.7* genes had the largest number of ABRE motifs, whereas *AsaDREB1.3*, *1.5*, *1.7*, *2.4*, and *2.6* had the largest number of MYB-binding sites (Table 2).

All of the analyzed genes contained motifs associated with JA that mediate plant development and stress response [40]: CGTCA motifs (except for *AsaDREB1.1*, *1.6–1.8*, *2.4*, and *2.6*), TCA elements (*AsaDREB1.6*, *1.8*, and *2.3*), or MYC TF-binding sites (except for *AsaDREB1.2*, *1.3*, *1.8*, and *2.7*); the largest numbers of the latter were detected in the *AsaDREB2.3*, *2.4*, and *2.6* promoters (Table 2). None of the genes contained SA-associated elements.

ET-responsive elements were found only in the promoters of *AsaDREB2* genes (except for *AsaDREB2.3* and *AsaDREB2.7*). Auxin and GA response-associated elements were identified in three (*AsaDREB1.2*, *2.3*, and *2.4*) and two (*AsaDREB1.3* and *2.3*) genes, respectively (Table 2).

Wounding- and pathogen-responsive elements (three different motifs) were identified in most genes (except *AsaDREB1.1*, *1.3*, *1.5*, and *2.7*). Among the abiotic stress-related elements, many were associated with anaerobic conditions, such as ARE (11 genes), and low temperature, such as LTR (7 genes). The STRE element associated with response to other abiotic stresses (heat, osmotic stress, low pH, and nutrient starvation) was more characteristic for the *AsaDREB1* genes (the largest number in *AsaDREB1.2*), whereas among the *AsaDREB2* genes, only *AsaDREB2.7* contained STRE. The least represented motif found only in *AsaDREB1.6* was related to drought response. TC-rich repeats and W-box involved in plant defense were found in the promoters of three (*AsaDREB1.3*, *1.6*, and *2.3*) and four (*AsaDREB1.3*, *1.5*, *2.1*, and *2.2*) genes, respectively (Table 2).

The promoters of two *AsaDREB1* and four *AsaDREB2* genes contained nitrogen-responsive motifs associated with endosperm-specific gene expression. *AsaDREB1.7*, *1.8*, and *2.5* had sites responsible for meristem-specific gene expression. Promoters of *AsaDREB2.5* and *2.8* contained bZIP and HD-Zip IV TF-binding sites, respectively (Table 2).

Most *AsaDREB1* genes (except *AsaDREB1.1* and *1.2*) had RY, re2f-1, or E2F-binding sites related to cell cycle regulation [41]. Among the *AsaDREB2* genes, *AsaDREB2.2* and *2.3* contained MSA-like elements (Table 2) involved in the cell-cycle-dependent promoter activation of mitotic B-type cyclin genes at the G2/M phase [42].

### 2.4. AsaDREB Expression in the Organs of Garlic Cultivars Ershuizao and Sarmat

**Figure 4:**
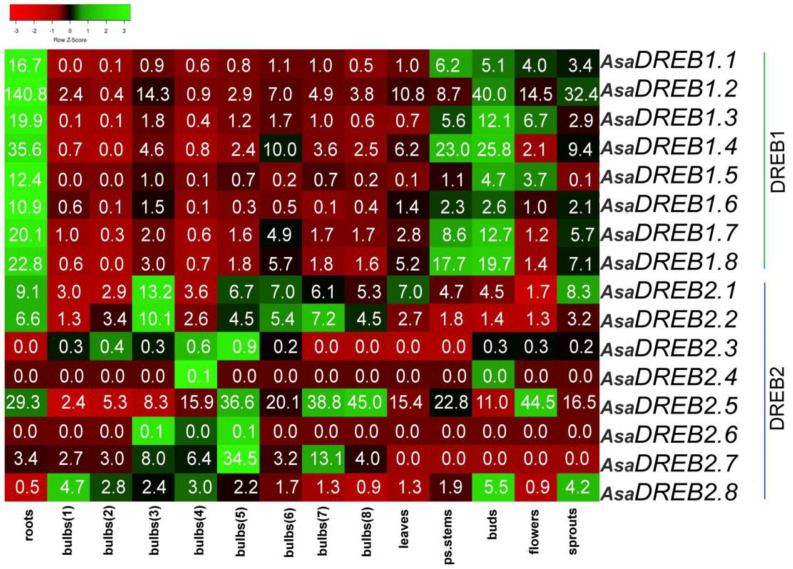
Heatmap of *AsaDREB1* and *AsaDREB2* expression in *A. sativum* cv. Ershuizao (PRJNA607255). *AsaDREB* mRNA levels were analyzed in the roots, bulbs (stages 1–8 corresponding to 192-, 197-, 202-, 207-, 212-, 217-, 222-, and 227-day-old bulbs, respectively), leaves, pseudostems (ps.stem), buds, flowers, and sprouts. The color scheme indicates the gene expression gradient from low (red) to high (green).

Analysis of the *A. sativum* cv. Ershuizao transcriptome (PRJNA607255) revealed that *AsaDREB1.1–1.8* and *AsaDREB2.1–2.8* genes were differentially expressed in various plant organs (Figure 4).

The results indicated that all *AsaDREB1* genes had high expression levels in the roots (10.9–140.8 RPKM) and buds (4.7–40.0 RPKM; except for *AsaDREB1.6* (2.6 RPKM)). Many genes were activated (>3 RPKM) in the leaves (*AsaDREB1.2*), pseudostems (all genes except *AsaDREB1.5*, and *1.6*), flowers (*AsaDREB1.1*, *1.2*, *1.3*, and *1.5*), and sprouts (all genes except *AsaDREB1.3*, *1.5*, and *1.6*). During bulb development from stages 1 to 8, the expression of the *AsaDREB1* genes increased significantly by stage 3, decreased sharply at stage 4, gradually increased at stages 5 and 6, and decreased at stages 7 and 8. In the analyzed garlic tissues, *AsaDREB1.2* had the strongest expression (except for the pseudostems and stage 5 bulbs), whereas *AsaDREB1.5* and *AsaDREB1.6* had the weakest (except for the roots) (Figure 4). In general, the *AsaDREB1* genes had similar tissue expression profiles, which were consistent with their clustering pattern on chromosome 1 (Figure 1a).

Among the *AsaDREB2* genes, tissue-specific expression was observed for *AsaDREB2.4* (only in stage 4 bulbs), *AsaDREB2.6* (stages 3 and 5 bulbs), *AsaDREB2.3* (stages 1–6 bulbs, buds, and flowers), and *AsaDREB2.7* (all-stage bulbs and roots). Only *AsaDREB2.7* had relatively high expression levels during bulb development, which fluctuated up and down from stage 5 to stage 8 (Figure 4).

The other *AsaDREB2* genes (*AsaDREB2.1*, *2.2*, *2.5*, and *2.8*) were expressed in all of the analyzed tissues; among these genes, the strongest expression was observed for *AsaDREB2.5* (maximum in the roots, flowers, and stage 4–8 bulbs, minimum in stage 1 and 3 bulbs). During bulb development, the mRNA levels of *AsaDREB2.1* and *AsaDREB2.2* fluctuated from stage 3 to 8, whereas those of *AsaDREB2.5* gradually increased throughout stages 1–5, sharply decreased at stage 6, and again increased at stages 7 and 8, and those of *AsaDREB2.8* gradually decreased from stage 1 to 8 (Figure 4).

**Figure 5:**
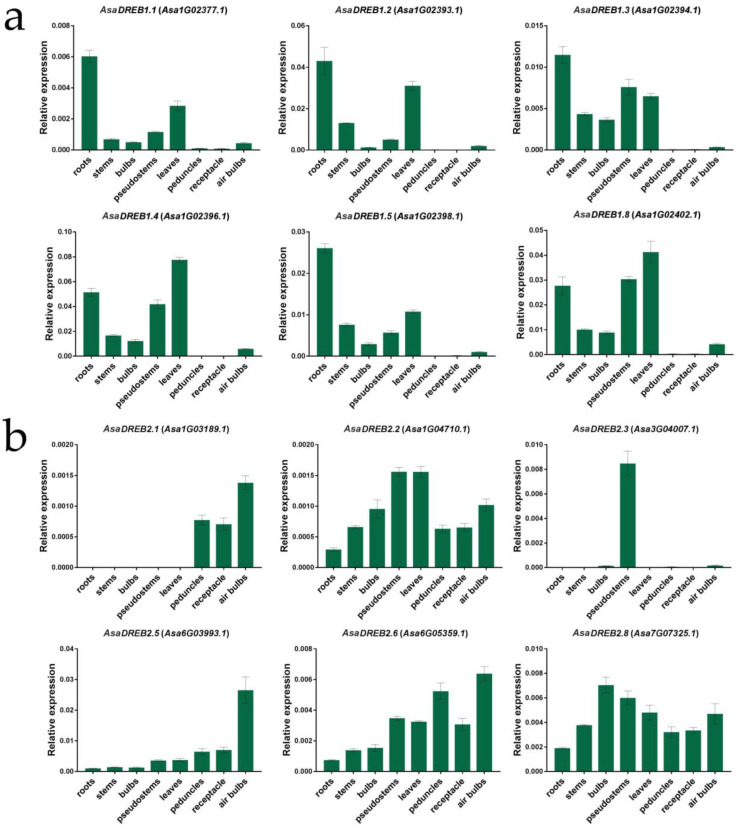
Transcription of the *AsaDREB1* and *AsaDREB2* genes in *A. sativum* cv. Sarmat; ps.stems, pseudostems. The data were normalized to *GAPDH* and *UBQ* mRNA levels. The significance of differences in the gene expression between organ types was analyzed using one-way ANOVA; obtained *p*-values are given in the Supplementary Table S2 (*p* < 0.05 indicates significant gene expression difference between organ types).

Next, we analyzed the transcription of the *AsaDREB genes* in the roots, stems, stage 8 bulbs, pseudostems, leaves, peduncles, receptacles, and air bulbs of *A. sativum* cv. Sarmat (Figure 5) using gene-specific primers (Supplementary Table S1).

The results indicated that the expression patterns of most *AsaDREB1* genes (*AsaDREB1.1–1.5* and *1.8*) in cv. Sarmat were similar to those in cv. Ershuizao. The two cultivars differed by the absence of *AsaDREB1.6* and *1.7* transcripts in all cv. Sarmat tissues and by the ratio of expression levels in specific organs. The strongest expression was observed in the roots (*AsaDREB1.1*, *1.3*, and *1.5*), leaves (*AsaDREB1.4*, and 1.8), or both (*AsaDREB1.2*) and the weakest—in the peduncles and receptacles. *AsaDREB1.1–1.5* and 1.8 had relatively low expression levels in the air bulbs, stems, and bulbs (Figure 5a).

Compared with the *AsaDREB1* genes, the *AsaDREB2* genes showed more significant inter-group transcriptional differences. Thus, in cv. Sarmat, transcripts of *AsaDREB2.1* were found only in the peduncles, receptacles, and air bulbs, and those of its closest structural homolog *AsaDREB2.2* were observed in all organs, with the maximum in the leaves and pseudostems and minimum in the roots (Figure 5b), whereas in cv. Ershuizao, the two genes had a similar expression pattern with the maximum in the roots (Figure 4). In cv. Sarmat, *AsaDREB2.3* transcription was strong in the pseudostems but weak in the bulbs, peduncles, and air bulbs (Figure 5b), whereas in cv. Ershuizao, it was not expressed in the pseudostems and stage 8 bulbs (Figure 4). *AsaDREB2.6* was transcribed in all analyzed organs (maximum in the peduncles and air bulbs and minimum in the roots; Figure 5b), but was practically not expressed in cv. *Ershuizao* (Figure 4). In cv. Sarmat, the *AsaDREB2.5* mRNA level in stage 8 bulbs was significantly lower than in the pseudostems and leaves (Figure 5b), whereas in cv. Ershuizao, the opposite pattern was observed (Figure 4). At the same time, in both cultivars, *AsaDREB2.3* transcription was absent in the roots and leaves, *AsaDREB2.4* was not transcribed in any organ, and *AsaDREB2.5* and *AsaDREB2.8* were expressed in all of the analyzed tissues (Figure 4 and Figure 5b).

### 2.5. AsaDREB1 and AsaDREB2 Expression in Garlic Seedlings in Response to Fusarium Infection

Considering the distinct patterns of hormone- and pathogen-responsive *cis*-regulatory elements in the promoters of the *AsaDREB1* and *AsaDREB2* genes (Table 2), we analyzed *AsaDREB* expression in the roots of *F. proliferatum*-infected garlic cultivars Sarmat and Strelets resistant and susceptible to FBR, respectively.

**Figure 6:**
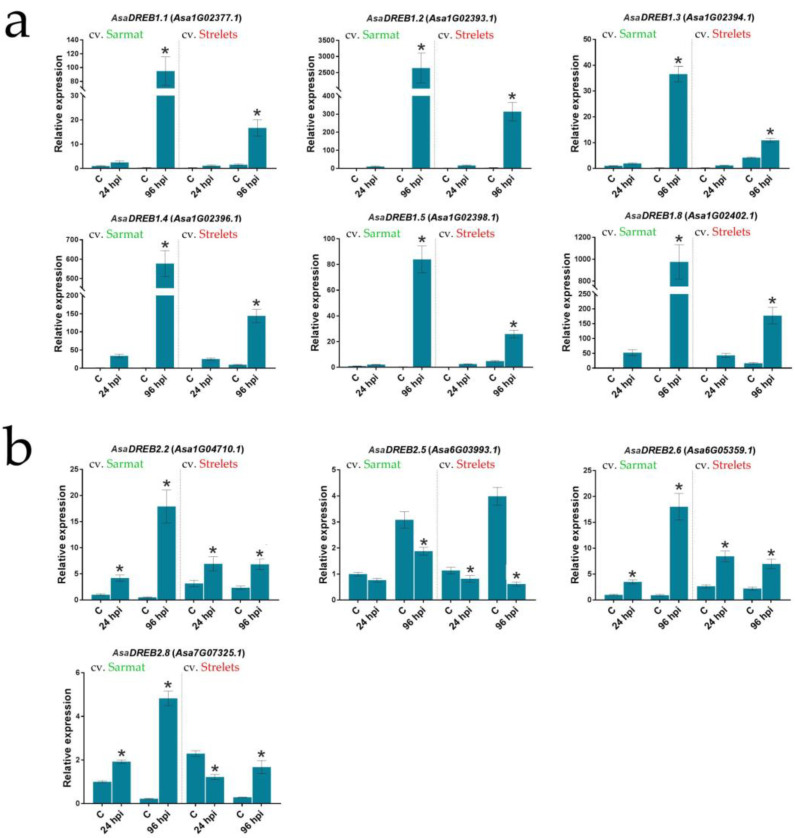
Expression of the selected *AsaDREB* genes in the roots of *A. sativum* FBR-resistant cv. Sarmat and FBR-susceptible cv. Strelets in response to *F. proliferatum* infection. The plants were incubated with *F. proliferatum* conidia and analyzed for the transcription of the indicated genes 24 and 96 h post inoculation (hpi). The data were normalized to *GAPDH* and *UBQ* mRNA levels and presented as fold change (mean ± SE) of control (expression in cv. Sarmat at 24 hpi taken as 1); * *p* < 0.05 compared with the uninfected control. The significance of differences in gene expression between the control and experiment, as well as between the time-dependent control and experiment data, was analyzed using one-way ANOVA; obtained *p*-values are given in Supplementary Table S3.

Among the *AsaDREB1* genes, *AsaDREB1.6* and *1.7* were not expressed, whereas all other genes were upregulated at 96 h post inoculation (hpi) in both cultivars, but the response in FBR-resistant cv. Sarmat was much stronger than in FBR-susceptible cv. Strelets. Thus, at 24 hpi, the mRNA levels of *AsaDREB1.1–1.5* and *1.8* were similar in the two cultivars, whereas at 96 hpi, the upregulation was 3.5–10 times greater in cv. Sarmat (35–2500 times vs. control) than in cv. Strelets (2.5–300 times) (Figure 6a).

Among the *AsaDREB2* genes, four (*AsaDREB2.2*, *2.5*, *2.6*, and *2.8*) were differentially regulated in response to *F. proliferatum* infection. *AsaDREB2.2* and *2.6* were upregulated and *AsaDREB2.5* was downregulated in both cultivars, whereas the *AsaDREB2.8* gene was upregulated at 24 and 96 hpi in cv. Sarmat but downregulated at 24 hpi and upregulated at 96 hpi in cv. Strelets (Figure 6b). Overall, the activation of the *AsaDREB2* genes was significantly stronger in the FBR-resistant than in the FBR-susceptible cultivar, whereas the inhibition (*AsaDREB2.5*) was weaker (Figure 6b).

Thus, in garlic, the magnitude of the *AsaDREB* transcriptional response to *F. proliferatum* infection appeared to depend on FBR susceptibility, although the pattern was the same for all of the genes (except *AsaDREB2.8*). Very similar infection response patterns of *AsaDREB1* genes (except *AsaDREB1.6* and *1.7*) (Figure 6a) corresponded to the clustering of these genes on chromosome 1 (Figure 1a). The close structural homology of *AsaDREB2.2* and *AsaDREB2.6* (Figure 2) was consistent with the same infection-induced transcriptional profiles, whereas the phylogenetically distant *AsaDREB2.5* (Figure 2) showed the opposite activation pattern (Figure 6b).

### 2.6. AsaDREB1 and AsaDREB2 Expression in Garlic Seedlings in Response to Cold Stress

**Figure 7:**
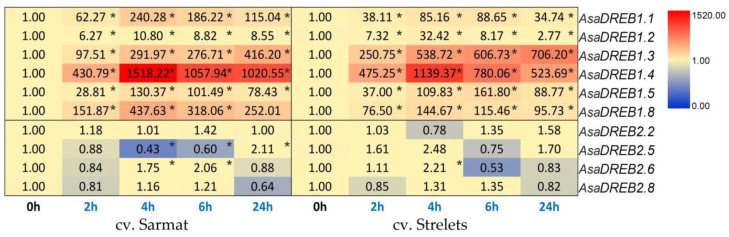
Heatmap of the time-dependent *AsaDREB* gene expression in *A. sativum* winter-hardy cv. Strelets and medium winter-hardy cv. Sarmat after cold stress. Plants were exposed to cold (+4 °C) for 2, 4, 6, and 24 h and analyzed for the expression of the indicated *AsaDREB* genes in the leaves. The data were normalized to *GAPDH* and *UBQ* mRNA levels; transcriptions at 0 h (normal conditions before treatment) were taken as 1. The color gradient indicated expression decrease (blue) and increase (red) relative to the control. * *p* < 0.01 compared with the control (0 h).

Given that cv. Strelets and Sarmat also differed in sensitivity to low temperatures (winter-hardy and medium winter-hardy, respectively), we analyzed the expression of the *AsaDREB* genes in the leaves of seedlings subjected to cold stress (+4 °C) for different times. Short-term exposure (2–6 h) had no effect on the leaf status, but after 24 h, slight wilting was observed in both cultivars. However, the effects on the *AsaDREB* transcription could be detected already at 2 h post treatment when all *AsaDREB1* and half of *AsaDREB2* genes showed a differential expression in response to cold (Figure 7 and Figure S3).

Thus, in both cultivars, significant activation of *AsaDREB1.1–1.5* and *1.8* genes (~6–430 times) was observed already at 2 h, which continued to increase after 4 h (~1.7–4.5 times) and remained strong thereafter, despite some decrease. The strongest transcriptional response was observed for *AsaDREB1.4* and the weakest for *AsaDREB1.2* (Figure 7).

In contrast, the *AsaDREB2* genes were mostly downregulated by cold (although slight activation (<2.5 times) was observed for some genes at certain time points), and the overall transcriptional response was much weaker than that of *AsaDREB1* (Figure 7). The *AsaDREB2* expression patterns were somewhat different in cv. Strelets and Sarmat; only *AsaDREB2.8* showed similar bell-shaped expression dynamics (Figure 7).

Among the 10 cold-responsive *AsaDREB* genes, only four (*AsaDREB1.5*, *1.8*, *2.2*, and *2.6*) contained low temperature-sensitive (LTR) *cis*-regulatory elements in the promoters (Table 2), and the transcriptional response of these genes was cultivar-dependent (Figure 7). Consistent with this pattern, *AsaDREB1.1–1.4*, and *AsaDREB2.8*, which did not contain LTR motifs (Table 2), had similar expression changes in both cultivars (Figure 7). At the same time, the *AsaDREB2.5* gene, which also did not have LTR motifs (Table 2), showed a cultivar-specific response to cold (Figure 7). As the analysis of the *cis*-element composition was based on cv. Ershuizao genes, it is quite possible that the *AsaDREB* genes of cv. Sarmat and Strelets could differ in their regulatory regions.

Overall, these results indicate that the *AsaDREB1* genes played an important role in the reaction of garlic to cold stress, whereas the involvement of the *AsaDREB2* genes was not obvious. The presence of LTR elements in the promoters of the *AsaDREB* genes was likely associated with the observed genotype-specific response of garlic to low temperatures.

## 3. Discussion

In this study, we aimed to identify and characterize *DREB* genes of the A1 and A2 subfamilies in the garlic genome. Overall, we found eight *DREB1* (A1) and eight *DREB2* (A2) genes in cv. Ershuizao through genome-wide analysis (Table 1). The number of *AsaDREB* genes was close to that in other plant species such as dicot *A. thaliana* (six A1 and eight A2 genes [38]) and monocots *Saccharum spontaneum* L. (Commelinids; twelve A1 and six A2 genes [37]) and *D. catenatum* (Asparagales; 26 *DREB* genes [36]). Considering that the high phenotypic variability of modern garlic was primarily due to the initial cross-pollination between fertile wild relatives in the center of origin [3,10,11], it can be assumed that the *DREB* gene family emerged in ancestral garlic accessions before the species lost fertility. Structural division of the AsaDREB proteins into A1 and A2 subfamilies (Figure 2) suggests their functional conservation, including the role in the plant defense response to stress.

We found that only 4 of the 16 identified genes contained introns: *AsaDREB2.1*, *2.2*, and *2.6* (1 intron) and *AsaDREB2.8* (3 introns) (Figure 1b). As it was believed that the intronless genes evolved from their intron-containing ancestors [43], this result suggested that *AsaDREB2.1*, *2.2*, *2.6*, and especially *AsaDREB2.8*, emerged earlier than the other identified *AsaDREB* genes.

The *AsaDREB1* genes form a tandem on chromosome 1 (Figure 1a), which may indicate their origin through tandem duplication of the precursor gene. This fact together with separate phylogenetic clustering of *AsaDREB1.1–1.8* proteins (Figure 2) suggested the functional redundancy of *AsaDREB1* in the transcriptional regulation in *A. sativum*. Consistent with this notion, we observed similar tissue expression patterns of the *AsaDREB1* genes (Figure 4 and Figure 5a) despite significant differences in the *cis*-regulatory element profiles (Table 2).

The *AsaDREB1.2* gene was expressed in cv. Ershuizao much stronger than the other *AsaDREB1* (Figure 4). *AsaDREB1.2* occupies a base position on the phylogenetic dendrogram in relation to the other *AsaDREB1 (*Figure 2) and is a possible precursor of the *AsaDREB1* cluster, carrying new valuable functions (compared with the earlier originated *AsaDREB2*). A common signature inherited from *AsaDREB1.2* and present in the other *AsaDREB1* genes includes a strong expression in the garlic roots and buds (Figure 4). Variations in the *AsaDREB1* expression levels in the other plant organs (Figure 4) may indicate functional changes in the A1 group. Thus, the increased expression of *AsaDREB1.4* and *1.8* in the pseudostem suggests their predominant regulatory activity in this tissue, whereas very low transcriptional levels of *AsaDREB1.5* in the seedlings, leaves, and bulbs (Figure 4) could be associated with the loss of the *AsaDREB1.5* regulatory function in these organs.

The differences in the *AsaDREB1* tissue expression profiles observed between cv. Ershuizao and Sarmat suggest garlic genotype-dependent functions of the *AsaDREB1* genes. Thus, in contrast with cv. Ershuizao, the expression of *AsaDREB1.2* was similar to that of *AsaDREB1.4*, *1.5*, and *1.8* (Figure 5a). At the same time, the activation of *AsaDREB1.4* and *1.8* in the pseudostems was preserved in cv. Sarmat (Figure 5a), suggesting that the neofunctionalization of the two genes is conserved in different garlic cultivars. The absence of *AsaDREB1.6* and *1.7* transcription in cv. Sarmat may indicate their genotype-specific pseudogenic nature.

Similar to *DREB1* genes of other plants [23,24,25,26,27,29], *AsaDREB1* genes (except for unexpressed *AsaDREB1.6* and *1.7*) were strongly activated by cold (Figure 7). Among them, the weakest induction was observed for *AsaDREB1.2*, which may indicate that the role in cold response acquired by this ancestral gene was later strengthened in the descendant *AsaDREB1* genes. Thus, the highest induction level of *AsaDREB1.4* in cv. Sarmat and Strelets (Figure 7) may indicate the predominant activity of this *AsaDREB1* gene in the cold response of garlic; the strong expression of the other two genes, *AsaDREB1.3* and *1.8*, in cold-treated plants also suggests their significant contribution to garlic protection against low temperatures. The cultivar-dependent expression of *AsaDREB1.3*, *1.4*, and *1.8* in response to cold treatment (Figure 7) could be associated with the difference in winter hardiness.

Our results indicated that the *AsaDREB1* genes could also be involved in the garlic defense against *F. proliferatum* infection, as evidenced by the much stronger activation of *AsaDREB1.1–1.5* and *1.8* in the FBR-resistant cv. Sarmat than in the FBR-susceptible cv. Strelets (Figure 6a). It should be noted that *AsaDREB1.2* had the highest expression level (Figure 6a), suggesting that the responsiveness to infection was acquired by the *AsaDREB1* genes relatively early in the evolution, but gradually weakened in the descendant genes. A still high activation level of *AsaDREB1.4* and *1.8* (Figure 6a) suggests that these genes, along with *AsaDREB1.2*, are closely involved in the reaction of garlic to fungal infection.

Cumulatively, our findings indicate that *AsaDREB1.2* is a precursor gene for the *AsaDREB1* subfamily in garlic, which carried cold and infection sensitivity traits to the *AsaDREB1* genes emerged later. The *AsaDREB1* genes are most active in the roots, which represent one of the stress-sensitive plant tissues; among these genes, *AsaDREB1.1–1.5* and *1.8* participate in plant response to cold stress and *F. proliferatum* infection. Three genes, *AsaDREB1.2*, *1.4*, and *1.8*, may perform most of the *DREB1* function in garlic, whereas the other *AsaDREB1* genes could play redundant roles.

The genes of the A2 subfamily are mainly scattered over different chromosomes (Figure 1a), which may indicate their origin through segmental gene duplications and suggest functional diversification. The latter notion is consistent with the distinct expression patterns of the *AsaDREB2* genes. *AsaDREB2.4* is not expressed in garlic, being probably a pseudogene, whereas the absence of *AsaDREB2.6* and *2.7* transcripts in one cultivar and the presence in the other (Figure 4, Figure 5b, Figure 6b and Figure 7) suggest a genotype-dependent pseudogene status of these *AsaDREB2* genes.

The *AsaDREB2* genes were transcribed in a tissue-specific manner both in cv. Ershuizao (Figure 4) and cv. Sarmat (Figure 5b), which may indicate their differential functional activity in garlic organs. The highest expression levels in all tissues of both cultivars were observed for *AsaDREB2.5* (Figure 4 and Figure 5b), suggesting its primary role in performing the DREB2 function in garlic, while the other *AsaDREB2* genes may complement *AsaDREB2.5* activity in specific organs and/or genotypes.

The expression profiles of some *AsaDREB2* genes (Figure 4 and Figure 5b) were consistent with their phylogeny (Figure 2). Thus, *AsaDREB2.1*/*2.2* and *AsaDREB2.4*/*2.6* had similar expression levels and were clustered together in the dendrogram; the two clusters were grouped, but with low bootstrap support. At the same time, *AsaDREB2.3*, *2.5*, and *2.7* with individual expression signatures were located distantly from the other *AsaDREB2* genes (Figure 2).

The closest homolog of the dominant *AsaDREB2.5* gene is *Arabidopsis* AT2G40340 (*DREB2C*) (Figure 2), known to be involved in plant response to abiotic stresses and hormones [44,45,46,47]. It is likely that the *AsaDREB2.5* gene plays similar roles in garlic, which would supplement its involvement in the response to cold and *Fusarium* infection (Figure 6b and Figure 7).

*AsaDREB2.2*, *2.6*, and *2.8* were significantly activated by *Fusarium* infection, whereas *AsaDREB2.1*, *2.3*, *2.4*, and *2.7* were not (Figure 6b), indicating the role of the former in the defense against fungal pathogens and the lack of it in the latter. At the same time, the expression of *AsaDREB2.2*, *2.6*, and *2.8* was affected by cold exposure, and although the response was much weaker than that of the *AsaDREB1* genes, it may indicate the contribution of *AsaDREB2.2*, *2.6*, and *2.8* to the cold resistance of garlic. However, the involvement of the *DREB2* genes in response to cold is still questionable [28,29,36] and requires further investigation.

Thus, *AsaDREB2.2*, *2.5*, *2.6*, and *2.8* are likely the main *AsaDREB2* genes that perform the *DREB2* function in garlic, among which *AsaDREB2.5* plays a dominant role in the response to biotic and abiotic stresses.

## 4. Materials and Methods

### 4.1. AsaDREB1 and AsaDREB2 Gene Subfamilies: Identification and Analysis

In this study, the genome (PRJNA606385) and transcriptomes (PRJNA607255) of *A. sativum* (cv. Ershuizao) [1] were used to search for full-length sequences of the *DREB1* and *DREB2* genes. Structural and phylogenetic analyses of the *AsaDREB1* and *AsaDREB2* genes were carried out in MEGA 7.0.26 [48]: the dendrogram was constructed using the Neighbor-Joining method; the evolutionary distances were computed by the JTT matrix-based method and were in the units of the number of amino acid substitutions per site; bootstrap values based on 1000 replicates were applied to evaluate the confidence for the tree topologies. The localization of *AsaDREB* genes on *A*, *sativum* chromosomes was visualized in MG2C v2.1 (http://mg2c.iask.in/mg2c_v2.1/; accessed on 3 February 2023). The *AsaDREB* exon–intron structures were predicted with GSDS v2.0 [49]. The domain structure of the proteins was characterized using the Conserved Domain Database (https://www.ncbi.nlm.nih.gov/cdd; accessed on 3 February 2023). The conserved motifs of the proteins were identified by MEME 5.5.1 (http://meme-suite.org/tools/meme; accessed on 3 February 2023), as well as considering the available published data. Protein MW and pI values were predicted with ExPASy ProtParam (https://www.expasy.org/resources/protparam; accessed on 3 February 2023). The PANNZER2 server (Protein ANNotation with Z-scoRE) was used for protein functional annotation in terms of Gene Ontology (GO) (http://ekhidna2.biocenter.helsinki.fi/sanspanz/; accessed on 3 February 2023).

### 4.2. AsaDREB1 and AsaDREB2 Gene Expression: In Silico Analysis

Transcriptomic data for the bulbs (8 developmental stages corresponding to 192, 197, 202, 207, 212, 217, 222, and 227 days after sowing (DAS), respectively), roots, leaves, pseudostems, buds, sprouts (217 DAS), and flowers (217 DAS) of *A. sativum* cv. Ershuizao (PRJNA607255) normalized as FPKM (fragments per kilobase of transcripts per million reads mapped) [1] were used for *AsaDREB1* and *AsaDREB2* gene expression profiling. The criterion for the reliability of the presence of gene transcripts was FPKM ≥ 10 in at least one of the analyzed types of organs. A heatmap of the gene expression was constructed and visualized with Heatmapper [50].

### 4.3. cis-Regulatory Elements in the Promoters of the AsaDREB1 and AsaDREB2 Genes: In Silico Search

*cis*-acting regulatory elements associated with developmental processes and responses to hormones and stress were searched for in the *AsaDREB1* and *AsaDREB2* ~1.0 kb region upstream of the start codon. This analysis was performed using a regularly updated PlantCARE database, which combines information on *cis*-elements, enhancers, and repressors in plant genes (http://bioinformatics.psb.ugent.be/webtools/plantcare/html/; accessed on 3 February 2023).

### 4.4. Garlic Cultivars and F. proliferatum Strain

In this study, we used accessions of winter garlic common in Russia: the medium-hardy cv. Sarmat and the winter-hardy cv. Strelets, resistant and susceptible to *Fusarium* infection, respectively. In October 2021, garlic was sown in the fields of the Federal Scientific Vegetable Center (Moscow region, Russia), and in May 2022, the plants were moved to greenhouse conditions (16 h photoperiod; 23 °C). After 2.5 months, individual samples (roots, bulbs, stems (basal plates), leaves, pseudostems, peduncles, receptacles, and air bulbs (bulbils)) were collected and used to analyze the tissue-specific gene expression.

The *F. proliferatum* strain used in the study was isolated in 2021 from infected cv. Strelets bulbs and stored in the collection of microorganisms (Research Center of Biotechnology of the RAS, Moscow, Russia). The test of the isolate for pathogenicity showed the appearance of rot on the garlic cloves 5 days after infection [19,20].

### 4.5. Stress Assays

The response to short-term cold stress was analyzed by placing garlic seedlings (3–4 leaf stage) into a climatic chamber (+4 °C) for 2, 4, 6, and 24 h; untreated plants were used as the control. The leaves (two biological replicates) were harvested at each time point and stored at −80 °C.

The garlic cloves were infected with *F. proliferatum* as previously described [20]. Peeled and sterilized (3 min in 70% ethanol, then washed with water) cloves of a uniform size were kept on wet filter paper at room temperature in the dark until active root growth (~72 h). The formed roots and germinated cloves were immersed in a suspension of *F. proliferatum* conidia (~10^6^ mL^−1^), blotted after 5 min, and transferred to fresh filter paper. Then, after 24 and 96 h (+25 °C, darkness), samples of roots, pseudostems, and cloves (*n* = 3 biological replicates) were collected; tissues of uninfected cloves were used as the controls. All of the samples were stored at −80 °C and were used for analysis of the gene expression.

### 4.6. AsaDREB Gene Expression Pattern in Garlic Cultivars: Quantitative (q) Real-Time (RT)-PCR Analysis

The collected garlic tissue samples (~0.1 g of each) were used for the total RNA isolation (RNeasy Plant Mini Kit and RNase free DNase set; QIAGEN, Hilden, Germany) followed by cDNA synthesis primed by oligo(dT) (GoScript Reverse Transcription System; Promega, Madison, WI, USA). RNA was qualified by gel electrophoresis. The concentration of nucleic acids was measured using a Qubit Fluorometer (Thermo Fisher Scientific, Waltham, MA, USA). Then, 3.0 ng of cDNA was taken for the gene expression analysis (qRT-PCR). The reaction also included SYBR Green RT-PCR mixture (Syntol, Moscow, Russia) and gene-specific primers. The reaction (95 °C 5 min and then 40 cycles (95 °C 15 s and 60 °C 40 s)) was run on a CFX96 Real-Time PCR Detection System (Bio-Rad Laboratories, Hercules, CA, USA).

Primers were designed by comparative analysis of *AsaDREB* mRNAs based on the cv. Ershuizao transcriptome [1] (Supplementary Table S1). Primer sequences were selected in the variable regions of the cDNA (forward and reverse primers were separated by at least one intron) and additionally validated using BLASTn (https://blast.ncbi.nlm.nih.gov/; accessed on 3 February 2023) and Primer3 (http://frodo.wi.mit.edu/primer3/, accessed on 3 February 2023). A mixture of cDNA preparations from different tissue samples at concentrations of 10.0, 1.0, and 0.1 ng per reaction was used to determine the efficiency and gene specificity of primers using qRT-PCR (three technical replicates). The criterion for the efficiency of a primer pair was a melting curve containing a single peak, which confirms the gain of only one amplicon during qRT-PCR. Testing showed 95–100% (R^2^ = 0.943–0.999) efficiency of the selected primers.

The *AsaDREB* gene expression was normalized to the combination of two reference genes encoding ubiquitin (*UBQ*; NCBI ID MZ171222.1) and glyceraldehyde 3-phosphate dehydrogenase (*GAPDH*; NCBI ID MZ171220.1) that were previously used separately in garlic [51,52] and onion (*Allium cepa* L.), respectively. The combination of two stably expressed references (*GAPDH* and *UBQ*) has been used to improve the accuracy of qRT-PCR analysis relative to single reference analysis [53], and has already shown its effectiveness in garlic studies [17,19,20].

### 4.7. Statistical Analysis of the AsaDREB Gene Expression Data

Statistical processing of gene expression analysis data (qRT-PCR) was carried out in GraphPad Prism version 9 (https://www.graphpad.com/scientific-software/prism/; accessed on 30 June 2023). On the graphs, the normalized expression data (considering three technical replicates of two biological replicates in each case) were presented as mean ± standard deviation (SD) (Figure 5, Figure 6 and Figure S3). In the case of Figure 7 and Figure S3, the statistical significance of the differences in the detected level of gene transcripts was estimated using an unpaired (independent) *t*-test, assuming that expression differs significantly at *p* < 0.01. In the case of Figure 5 and Figure 6, the statistical significance of differences in the detected level of gene transcripts was estimated using one-way ANOVA (multiple comparisons, corrected with Bonferroni test); obtained *p*-values are given in Supplementary Tables S2 and S3, respectively. For Figure 5, the mean of each column was compared to the mean of every other column. For Figure 6, the means of the preselected pairs of columns (control (C) 24 h vs. experiment (E) 24 hpi; C 96 h vs. E 96 hpi; E 24 hpi vs. E 96 hpi; C 24 h vs. C 96 h; for each of two analyzed garlic cultivars) were compared. All *p*-values are given in Supplementary Table S2 (for Figure 5), Table S3 (for Figure 6) and Table S4 (for Figure 7).

## 5. Conclusions

We identified and characterized eight *DREB1* and eight *DREB2* subfamily genes in the *A. sativum* cv. Ershuizao genome and compared their organ-specific expression patterns with those in cv. Sarmat. Our results suggest that the tandemly clustered *AsaDREB1* genes could have redundant functions in transcriptional regulation in garlic; among them, *AsaDREB1.2*, *1.4*, and *1.8* may be the main genes performing the *DREB1* function in garlic, whereas *AsaDREB1.6* and *1.7* could be pseudogenes depending on the genotype. The *AsaDREB1* genes are most active in the roots and are stimulated by cold stress and *Fusarium* infection, suggesting their role in garlic protection against abiotic and biotic stresses. The *DREB2* subfamily genes *AsaDREB2.2*, *2.5*, *2.6*, and *2.8* are likely the functionally dominant *DREB2* genes in garlic, among which *AsaDREB2.5* has the highest expression level. The transcription of these four *AsaDREB2* genes is affected by low temperatures and *Fusarium* infection, suggesting their contribution to garlic adaptive responses. *AsaDREB2.4* could be a pseudogene, whereas the pseudogenic status of *AsaDREB2.6* and *2.7* may be genotype-specific. The identification and characterization of the *DREB* genes in garlic performed in this study provides the foundation for further functional analysis of the DREB TFs in *A. sativum* and the other *Allium* spp., and could contribute to the breeding of stress-tolerant cultivars.

## Figures and Tables

**Table 1:** Characteristics of the *DREB1.1–1.8* and *DREB2.1–2.8* genes in the *A. sativum* cv. Ershuizao genome.

Gene Name	Gene/Transript ID [1]	Genomic Localization	Gene, bp	CDS, bp	Protein, aa	MW, kDa	pI
*DREB1* subfamily							
*AsaDREB1.1*	Asa1G02377.1/Asa2G02428.1	chr1:643013697-643014305	609	609	202	22.68	5.35
*AsaDREB1.2*	Asa1G02393.1/Asa2G02432.1	chr1:647143533-647144066	534	534	177	19.12	4.70
*AsaDREB1.3*	Asa1G02394.1/Asa2G02430.1	chr1:647572970-647573575	606	606	201	22.44	5.03
*AsaDREB1.4*	Asa1G02396.1/Asa2G02427.1	chr1:648016507-648017130	624	624	207	23.00	5.31
*AsaDREB1.5*	Asa1G02398.1/Asa2G02425.1	chr1:649128626-649129231	606	606	201	22.34	5.18
*AsaDREB1.6*	Asa1G02399.1/Asa2G02422.1	chr1:649153916-649154545	630	630	209	23.11	5.77
*AsaDREB1.7*	Asa1G02401.1/Asa2G02424.1	chr1:649317409-649318038	630	630	209	23.11	5.77
*AsaDREB1.1*	Asa1G02377.1/Asa2G02428.1	chr1:643013697-643014305	609	609	202	22.68	5.35
*DREB2* subfamily							
*AsaDREB2.1*	Asa1G03189.1/Asa2G01510.1	chr1:865525192-865528432	3241	870	289	32.67	4.97
*AsaDREB2.2*	Asa1G04710.1/Asa0G03205.1	chr1:1262498222-1262501613	3392	870	289	32.63	5.29
*AsaDREB2.3*	Asa3G04007.1/Asa1G05763.1	chr3:1103416519-1103417280	762	762	253	28.07	6.11
*AsaDREB2.4*	Asa5G04281.1/Asa5G01946.1	chr5:1152951019-1152951420	402	402	133	14.98	9.91
*AsaDREB2.5*	Asa6G03993.1/Asa6G04032.1	chr6:1068494972-1068498718	3746	837	278	30.57	4.86
*AsaDREB2.6*	Asa6G05359.1/Asa6G02115.1	chr6:1450079651-1450080070	420	420	139	15.55	9.69
*AsaDREB2.7*	Asa7G07291.1/Asa5G06740.1	chr7_2:939903143-939903742	600	600	199	21.65	4.93
*AsaDREB2.8*	Asa7G07325.1/Asa5G06785.1	chr7_2:949089009-949094185	5177	450	149	16.86	9.76

**Table 2:** *cis*-elements found in the *AsaDREB1* and *AsaDREB2* regulatory regions (~1000 bp).

Motif Name	Annotation	*DREB1*								*DREB2*							
		*AsaDREB1.1*	*AsaDREB1.2*	*AsaDREB1.3*	*AsaDREB1.4*	*AsaDREB1.5*	*AsaDREB1.6*	*AsaDREB1.7*	*AsaDREB1.8*	*AsaDREB2.1*	*AsaDREB2.2*	*AsaDREB2.3*	*AsaDREB2.4*	*AsaDREB2.5*	*AsaDREB2.6*	*AsaDREB2.7*	*AsaDREB2.8*
Hormone response																	
ABRE	ABA-responsive	4	2	8	3	4	2			2	2	1	2	4	1	5	2
CARE													1		1		
TGA-element	Auxin-responsive		1									1	1				
CGTCA-motif	JA-responsive		1	1	1	1				2	2	1		1		1	2
TCA-element							1		1			1					
P-box	GA-responsive			1								1					
ERE	ET-responsive									1	1		1	1	1		1
Stress response																	
ARE	Essential for the anaerobic induction		4		1	1	1			5	5	1	1	1	1	2	
DRE1/DRE core	Drought responsive						1										
LTR	Low-temperature response					1		2	4	1	1		1		1		
STRE	Response to heat, osmotic stress, low pH, and nutrient starvation	2	5	1	1	2										3	
TC-rich repeats	Defense and stress response			1			1					1					
W-box	WRKY binding site; defense and biotic and abiotic stress response			1		1				1	1						1
Wun-motif	Wounding and pathogen response				1			1	1	1	1	2	1	1	1		
WRE3			1				1	2	2	1	1	1					
Box S																	1
Developmental processes																	
O2-site	Nitrogen-responsive; endosperm-specific gene expression		3				1						1	1		1	
GCN4 motif												1					
CCGTCC motif	Meristem-specific gene expression							1	1					1			
RY-element	Seed-specific gene expression			1		1											
MSA-like	Cell cycle regulation										1	1					
Other *cis*-elements																	
CCAAT-box/MYB/MRE/MBS1	MYB-binding site	2	2	5	1	7	1	4	3	3	3	2	5	2	5	2	3
Re2f-1	E2F-binding sites; seed-specific, cell-cycle regulated gene expression	1	1														
E2Fb					1		1	1	1								
MYC	MYC-binding site	1			1	1	1	1		1	1	4	3	1	4		1
CTAG-motif	Unknown	1								1	1						
A-box (TACGTA)	bZIP-binding site													1			
HD-Zip IV	HD-Zip IV-binding site																1

Color intensity (pale to dark) corresponds to the number of *cis*-elements (low to high).

## Data Availability

*AsaDREB1.1–1.8/2.1–2.8* sequences are available in the NCBI database (see Table 1).

## Supplementary Materials

The following supporting information can be downloaded at: https://www.mdpi.com/article/10.3390/plants12132538/s1, Table S1: List of primers for *AsaDREB* gene expression analysis; Table S2: Differences (*p*-values) in the expression level of the *AsaDREB* genes between different garlic organs; Table S3: Differences (*p*-values) in the expression level of the *AsaDREB* genes in the roots of *A. sativum* FBR-resistant cv. Sarmat and FBR-susceptible cv. Strelets in response to *F. proliferatum* infection compared to uninfected control; Table S4: Differences (*p*-values) in the expression level of the *AsaDREB* genes in the roots of *A. sativum* cv. Sarmat and cv. Strelets in response to cold stress compared to unstressed control; Figure S1: Alignment of the *AsaDREB1.1–1.8* (**a**) and *AtDREB2.1–2.8* (**b**) proteins with their homologs from *Arabidopsis thaliana* (NCBI IDs are indicated). Gray-shaded regions are 70–100% identical, red frame indicates AP2 domain. Consensuses typical for DREB proteins are underlined; Figure S2: Evolutionary relationships among DREB proteins from *Allium sativum* (blue), *Arabidopsis thaliana* (red), and *Zea mays* (green). The unrooted dendrogram was constructed using the Neighbor-Joining method (bootstrap test: 1000 replicates) in MEGA 7.0.26; Figure S3: Expression of the selected *AsaDREB* genes in the leaves of *A. sativum* cv. Strelets and cv. Sarmat after cold stress (+4 °C for 2, 4, 6, and 24 h). The data were normalized to *GAPDH* and *UBQ* mRNA levels; * *p* < 0.01 compared to control (0 h).
